# GibbsCluster: unsupervised clustering and alignment of peptide sequences

**DOI:** 10.1093/nar/gkx248

**Published:** 2017-04-12

**Authors:** Massimo Andreatta, Bruno Alvarez, Morten Nielsen

**Affiliations:** 1 Instituto de Investigaciones Biotecnológicas, Universidad Nacional de San Martín, 1650 San Martín, Argentina; 2 Department of Bio and Health Informatics, Technical University of Denmark, DK-2800 Lyngby, Denmark

## Abstract

Receptor interactions with short linear peptide fragments (ligands) are at the base of many biological signaling processes. Conserved and information-rich amino acid patterns, commonly called sequence motifs, shape and regulate these interactions. Because of the properties of a receptor-ligand system or of the assay used to interrogate it, experimental data often contain multiple sequence motifs. GibbsCluster is a powerful tool for unsupervised motif discovery because it can simultaneously cluster and align peptide data. The GibbsCluster 2.0 presented here is an improved version incorporating insertion and deletions accounting for variations in motif length in the peptide input. In basic terms, the program takes as input a set of peptide sequences and clusters them into meaningful groups. It returns the optimal number of clusters it identified, together with the sequence alignment and sequence motif characterizing each cluster. Several parameters are available to customize cluster analysis, including adjustable penalties for small clusters and overlapping groups and a trash cluster to remove outliers. As an example application, we used the server to deconvolute multiple specificities in large-scale peptidome data generated by mass spectrometry. The server is available at http://www.cbs.dtu.dk/services/GibbsCluster-2.0.

## INTRODUCTION

Peptide ligands are short amino acid sequences that play fundamental roles in countless biological processes, from molecular signaling to the regulation of immune responses. Even when receptors are activated by full proteins, linear components of the interaction with the ligand can often be modeled with short peptides (1). Conserved sequence motifs ensure high receptor specificity toward their cognate ligands, while avoiding as much as possible interference by unspecific ligands. Binding specificity often manifests itself as multiple distinct motifs, i.e. a poly-specificity of a single receptor (2). For instance, peptide recognition domains such as SH3 and PDZ domains are known to accommodate their binders in alternative conformations (3). Multiple specificities can also emerge as a result of assays interrogating several receptors in a single experiment, for example in systems where several receptor isoforms are expressed *in vivo* at the same time (4) and the corresponding ligand pool will describe a mixture of multiple motifs. Identifying the sequence motifs driving these interactions takes on additional levels of complexity if the motifs are located at different locations within the peptides (i.e. they require an alignment), or if they have heterogeneous length (i.e. they contain insertions or deletions (indels)).

The GibbsCluster server was created to deal with the challenges mentioned above, aiding the identification of motifs in peptides datasets that are in general unaligned, consist of multiple specificities and may contain indels. In simple terms, the program takes as input a set of peptide sequences and clusters them into meaningful groups. Sequence alignment and clustering are performed simultaneously by sampling the space of possible solutions using a Gibbs sampling strategy. Each cluster is represented by a position-specific scoring matrix (PSSM), and the algorithm aims to maximize the information content of individual matrices while minimizing the overlap between distinct clusters. Note that the algorithm detects at most one motif occurrence per peptide, hence assigning each peptide unequivocally to only one cluster. A trash cluster captures outliers that cannot be clustered with other peptides and is useful to filter out noise from raw data. The server returns a detailed report on the optimal clustering solutions, including plots of the optimal number of clusters and graphical representations of the identified sequence motifs as sequence logos.

Since the first GibbsCluster release and publication (5), the method has been used in several independent studies, in particular to deconvolute multiple specificities in major histocompatibility complex (MHC) class I peptidome datasets (6–8) but also on MHC class II data (9,10) and to aid the identification of post-translational modification sites (11). Large-scale studies of naturally presented MHC ligands, enabled by recent advances in mass-spectrometry, generate extensive datasets of potential T cell epitopes. Albeit the MHC genotype of a given cell line can be determined, the MHC restriction of individual ligands is generally unknown. On this kind of immuno-peptidomics data, GibbsCluster has gained increasing attention due to its ability to cluster peptides into multiple specificities and in this way infer their MHC restrictions. Ritz *et al.* (7) found that consensus motifs produced by GibbsCluster on 9-mer ligands from several cell lines showed ‘excellent agreement with published HLA binding motifs.’ Mommen *et al.* (9) compared the binding motifs of three HLA-DR molecules made by GibbsCluster to the motifs derived with a predictor explicitly trained on peptide-MHC binding data (NetMHCIIpan), and concluded that the unsupervised clustering gave solutions reasonably similar to the NetMHCIIpan motifs.

The previous version of the method was limited to continuous, ungapped sequence alignments. Because of the nature of the MHC-I molecule, closed at both ends and binding long ligands by means of a central bulge (12), this restriction effectively limited deconvolution analyses of MHC-I ligands to peptides of a fixed length, typically 9-mers. Insertions and deletions (indels), now implemented as part of GibbsCluster version 2.0, lift this important limitation. Inspired by earlier work, binding of ligands with non-conventional binding-core length is modeled using single insertions (to model ligands with short binding-core length) and deletions (to model ligands with extended binding-core length) (13,14). Analyzing a large set of MHC-I ligands of different lengths, we demonstrate how these can be effectively clustered and analyzed using the updated GibbsCluster method and that the clustering reveals allele-specific preferences in terms of peptide length. With the updated GibbsCluster server we provide a simple, effective tool to detect multiple sequence motifs in peptide datasets. In this paper, we describe the web interface, its output and provide an example of usage.

## WEB INTERFACE

### Submission page

#### Input data

The essential input to the server is a list of peptide sequences in standard one-letter amino acid code. An optional label (text without spaces) can be included as a second column in the input data. The labels are carried over to the results, and may be useful for correlating a known classification with the clustering solution produced by the method. The submission page includes examples of peptide data, labeled and unlabeled, and a button to automatically upload sample data.

#### Options and parameters

Several parameters can be specified to customize the clustering analysis. A number of recommended parameter configurations for specific problems can also be automatically uploaded by clicking on dedicated buttons. Basic options include a textual identifier for the run, the motif length and the number of clusters. If the latter is provided as an interval (e.g. 1–8), the method will suggest the optimal number of clusters as part of the output. The parameter λ (penalty for inter-cluster similarity) specifies how similar different clusters are allowed to be. For data containing multiple specificities with well-defined motifs, λ can be relatively high (e.g. λ = 1); on the other hand, if the aim is to detect subtle differences in mostly homogenous data, the parameter λ should be set to a lower value (e.g. λ = 0.2). The weight on small clusters σ determines whether clusters composed of few sequences should be allowed or penalized in favor of larger, more general clusters. The mathematical formulations of λ and σ are described in detail elsewhere (5). Gibbs sampling is a heuristic rather than a deterministic optimization procedure. Therefore, it cannot guarantee that the optimal solution is always reached from any starting configuration. A common procedure to boost performance is to repeat the sampling from a number of initial random configurations and select the solution that appears to be optimal in terms of the fitness function that governs the system. The number of initial seeds for multiple sampling can be specified as a parameter for the server. The cooling schedule can be customized by specifying initial Monte Carlo temperature, number of cooling steps and relative frequency of the various moves of the algorithm. By enabling the ‘trash cluster’, GibbsCluster can also automatically filter outliers that do not match to any of the clusters. This option can be very useful to remove potential noise from the input data. If the feature is selected, a scoring threshold for placing data into the trash cluster can be customized. The methodological details and mathematical formulation of the algorithm are discussed thoroughly in Andreatta *et al.* (5) and are substantially unchanged in version 2.0. The essential new feature of GibbsCluster-2.0 is the implementation of insertions and deletions, enabling the generation of gapped sequence alignments. Indel moves (attempts to introduce an insertion or a deletion at any possible position of a given peptide), are performed at an interval of iterations specified by the user, selecting the solution with the highest peptide score. A detailed description of the options, including guidelines and a basic glossary, can be accessed by clicking on the ‘Instructions’ tab of the main server page.

### Output page

**Figure 1.**
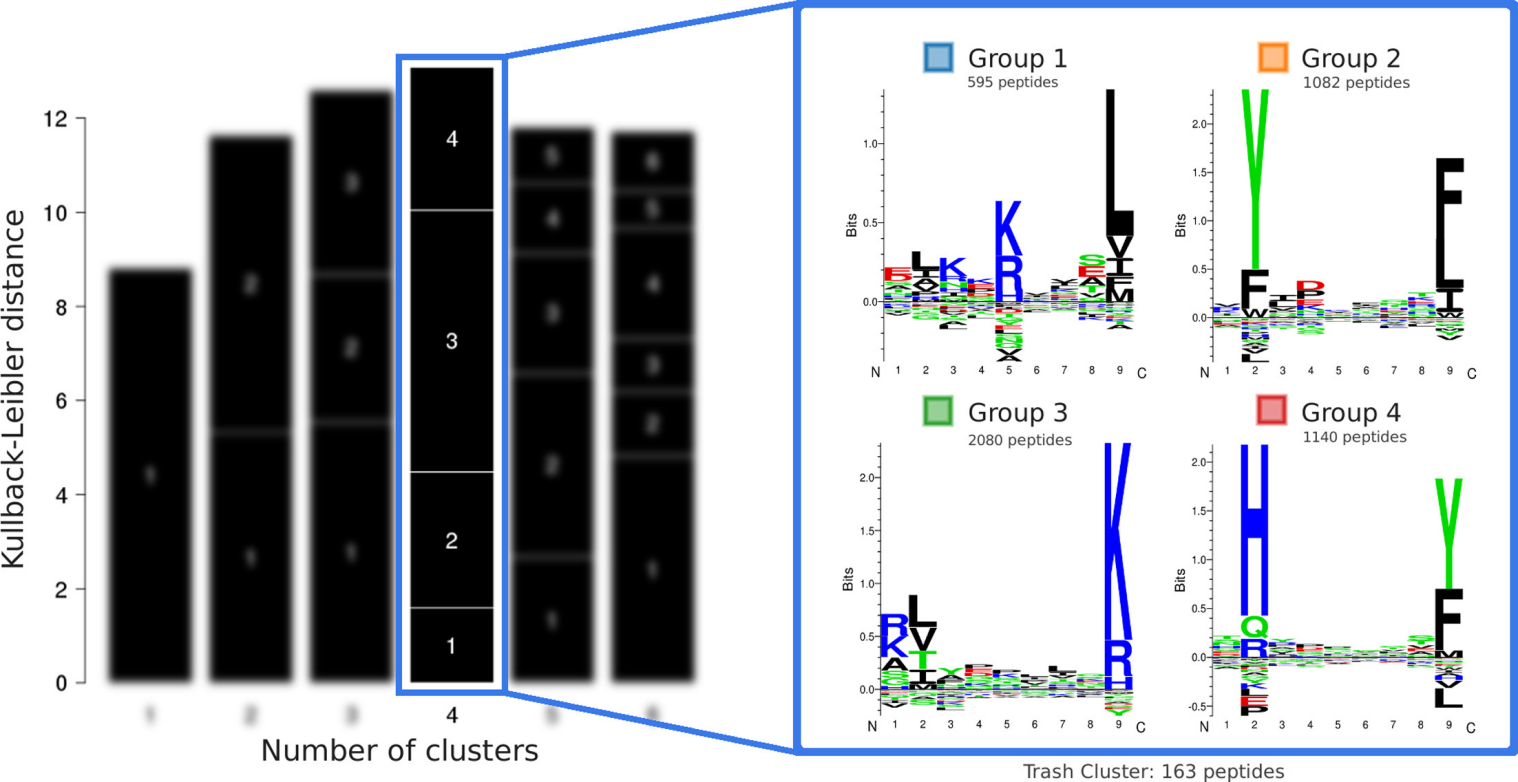
Clustering results for the Fibroblast dataset. The solution with highest KLD consists of four clusters, and the corresponding sequence motifs are shown as sequence logos.

The preamble of the output page includes a summary of the input data and the parameters specified for the run. If a name was assigned to the job, it will be reported here. Next, a barplot of the Kullback-Leibler Distance (KLD) as a function of the number of clusters suggests the optimal number of motifs in the data (Figure 1, left). The relative size of each black block within a bar is proportional to the size of each of the clusters. To the side of the barplot, the sequence motifs derived from the best solution (i.e. the solution with highest KLD) are displayed in the form of sequence logos generated with Seq2Logo (15). In the example of Figure 1, the algorithm detected four clusters with markedly different sequence motifs, with group 1 being the smallest and group 3 the largest.

Because the ‘optimal’ number of cluster depends at least partially on the job parameters and clustering granularity required by the individual user, alternative solutions may be relevant for the problem at hand. For this reason, the clustering solutions and motifs for each initial number of clusters are all included in the output page. Links to full alignment and clustering files in plain text provide detailed information about the group assignment, scores and alignment core of each peptide. The sequence motifs for each cluster can be inspected both in form of a PSSM and as sequence logos. Clicking on the LOGO button automatically transfers the peptide data to the Seq2Logo server, allowing further customization of the logos including alternative color schemes and different logo types. Finally, a link for bulk download at the bottom of the results page generates a compressed folder containing all the results, including plots, sequence logos and alignment/clustering files. A complete description of file formats can be found at the ‘Output format’ link from the main server page.

## EVALUATION AND CASE STUDY

To illustrate a typical application, we employed the GibbsCluster server to identify multiple specificities in HLA-I peptidomics data. The dataset consists of naturally presented ligands detected by mass spectrometry in six different cell lines (6). Before submitting the data to GibbsCluster, ligands of length outside the range 8–11 were excluded. GibbsCluster was run with a motif length of 9, allowing up to 2 deletions and up to 1 insertion, specifying an interval of 1–6 clusters. Because HLA-I ligand data are nearly aligned and mostly composed of 9mers, we recommend activating the option ‘make clustering move at each iteration’. The trash cluster was used to remove all peptides with a score lower than 2, and each clustering job was repeated from 5 alternative initial random configurations. All other parameters were left at default value.

**Figure 2.**
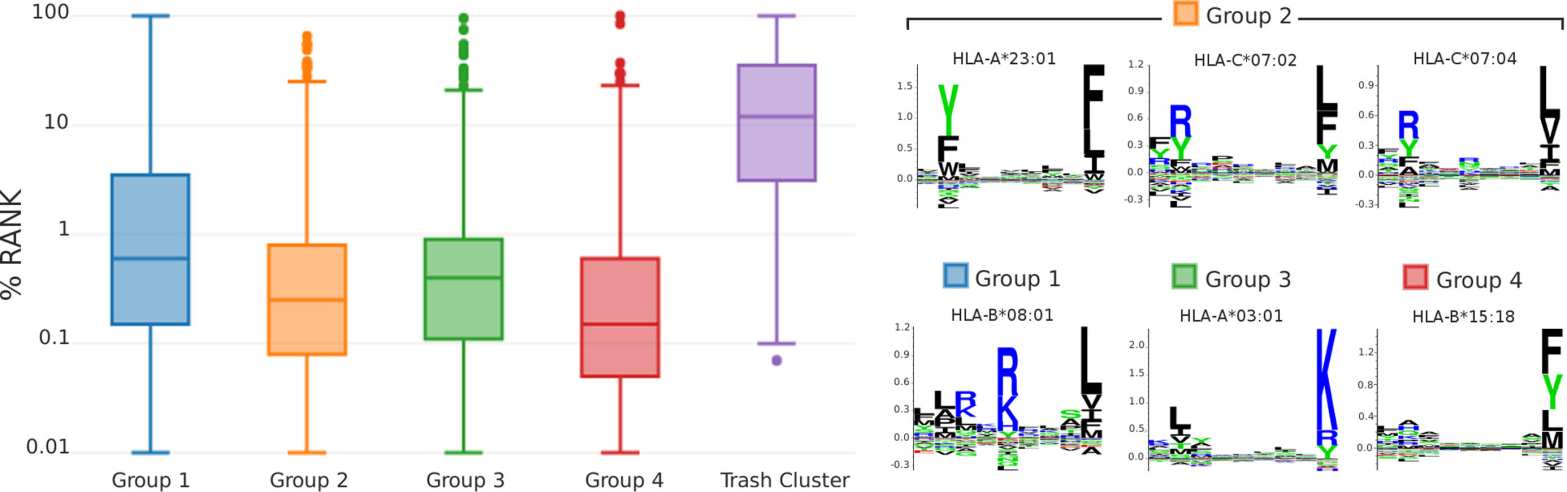
Comparison of unsupervised clustering to HLA restrictions assigned by NetMHCpan on Fibroblast data. Left: distribution of percentile rank scores predicted by NetMHCpan for the allele dominating each cluster; for the trash cluster, the best predicted rank score to any of the six alleles was used. Right: sequence logos from literature (made with MHCcluster (16)) of the alleles found in each cluster; group 2 is composed mostly of ligands predicted by NetMHCpan to be restricted to three different alleles with similar binding motifs.

The optimal solution identified by GibbsCluster for the Fibroblast cell line consisted of four clusters (Figure 1), with group 1 being the smallest (595 peptides) and group 3 the largest (2080 peptides). The four motifs have distinct and conserved preferences mostly at P2 and P9, with group 1 showing also an informative P5. On different cell lines, the method generally identified between 2 and 4 different clusters (Supplementary Figure S1). While the HLA restriction of individual ligands is *a priori* unknown, the HLA genotype of each cell line had been determined by Bassani-Sternberg *et al.* (6). NetMHCpan-3.0 rank prediction scores (14) were used to assign the most likely restriction of each ligand among the HLAs expressed in a given cell line. Based on these rank values, the dominant allele of each cluster can be assigned using a majority vote. For the Fibroblast dataset, the median percentage rank predicted by NetMHCpan to the dominant cluster allele is lower than 1% for all groups (Figure 2). In general peptides with predicted binding rank values <2% are considered MHC binders (13). In contrast, the majority of ligands assigned to the trash cluster have rank >10% to all expressed HLAs and are therefore likely to be incorrect measurements.

Looking at the composition of each cluster in terms of known MHC alleles, we observed a remarkable correspondence between the motifs identified by GibbsCluster and HLA binding motifs from literature (Figure 2, right). However, predicted binders to one HLA-A and to two HLA-C alleles were all clustered together into group 2. A total of 47% of the ligands in this cluster were predicted by NetMHCpan to bind to all these three molecules with rank <2 and 70% to at least two molecules. Because these three alleles have very similar binding preferences, the method cannot separate the three motifs and the six HLAs expressed in this cell line result in only four separate clusters. As noted previously (6,17) and confirmed on other cell lines (Supplementary Figure S2), unsupervised clustering tends to underestimate the number of specificities when multiple HLAs have highly overlapping motifs, especially in the case of HLA-C alleles, which have low expression levels and redundant motifs (18). Overall these results are in line with the recent findings by Bassani-Sternberg and Gfeller (17), who applied an unsupervised approach based on mixtures of weight matrices to deconvolute HLA I peptidomes. Like the GibbsCluster, this mixture model does not rely on MHC binding predictions to assign HLA restriction. However, also using this approach, complete deconvolution remains unachievable in situations where alleles have very similar motifs. The work by Bassani-Sternberg and Gfeller suggests a solution to this problem by extending the motif search to include additional unique motifs contained within suboptimal KLD cluster solutions. Applying a similar approach and investigating suboptimal KLD cluster solutions obtained with GibbsCluster allows identification of additional binding motifs. For instance, doing this for the HCT166 dataset allowed us to identify five distinct clusters with motifs corresponding to HLA-A*01:01, HLA-A*02:01, HLA-B*1801, HLA-B*4501 and HLA-C*0501 molecules expressed by the cell line (data not shown), suggesting that some additional visual and analytic analyses of the GibbsCluster results beyond the automated KLD-defined optimal solution in some cases can be beneficial. Beyond this, it is important to underline two important differences between the two methods; namely that the GibbsCluster method can handle peptides of variable length, and that GibbsCluster is publicly available as a web server.

**Figure 3.**
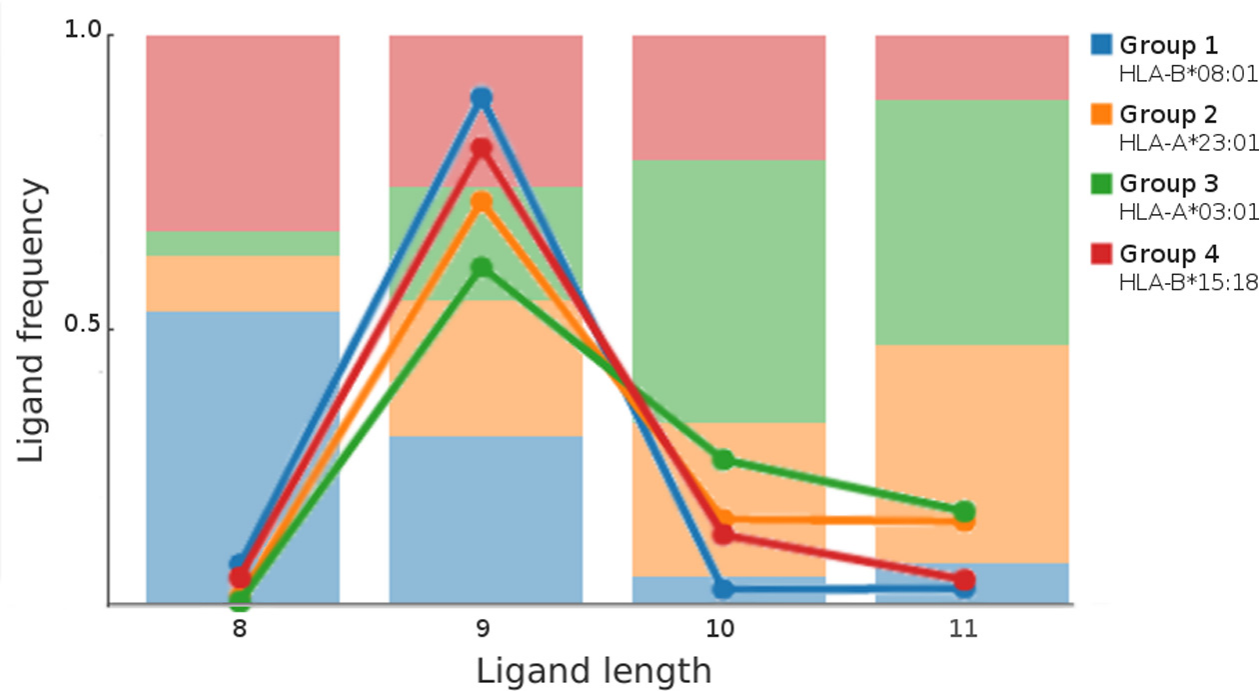
Length profile of peptides in the optimal Fibroblast clustering solution. Solid lines represent, for each group, the percentage of peptides with a given length over the total number of peptides in the group. The stacked bar plot in the background is the corresponding length frequency (number of ligands of a given length in a given group divided by the total number of peptides of that length in all groups).

As previously reported (19), MHC molecules exhibit distinct preferences in terms of ligand length. While in most cases 9mer peptides are largely favored over other lengths, binding preferences are skewed toward shorter or longer ligands for different alleles. The ability to cluster peptides of variable length allows the GibbsCluster to study these length preferences. In the optimal solution generated by GibbsCluster for the Fibroblast dataset, group 3 (mostly composed of HLA-A*03:01 ligands) is relatively enriched in 10mer and 11mer peptides, while it contains close to no 8mers. Group 1 (corresponding with the majority of HLA-B*08:01 ligands) is composed mostly of 9mers, but has the largest relative fraction of 8mers of the four clusters (Figure 3). Length preferences were also clearly observed on other cell lines characterized by different sets of HLA alleles (Supplementary Figure S3).

## DISCUSSION

Clustering and alignment of amino acid sequences is an optimization problem with a very large search space and a solution landscape characterized by many local optima. Gibbs sampling is an efficient heuristic approach to navigate such complex landscapes and approach the globally optimal solution (20). Compared to other methods for motif discovery, GibbsCluster is unique because it implements sequence alignment and clustering as alternative sampling moves, simultaneously constructing sequence alignments and clusters with distinct motifs. Deconvoluting ligand sequences into multiple motifs can be extremely useful as a guide to interpret the biological underpinning of receptor-ligand interactions, both when the system under study contains multiple receptor variants or when a single receptor accommodates multiple modes of binding.

As a case study, we reported the application of GibbsCluster to the deconvolution of HLA-I peptidomes generated by mass-spectrometry for several cell lines. While GibbsCluster has been previously employed to analyze this kind of high-throughput data (e.g. (7)), its applications were in these situations limited to binding motifs of a fixed length. The introduction of insertions and deletions in the sequence alignments generated by the method lifted this restriction and allowed us to generate accurate clustering solutions and binding motifs from HLA-I ligands of variable length. A simple example running the GibbsCluster method with and without indels on a set of 500 HLA-A01:01 restricted ligands of length 8–11 can illustrate the impact of lifting this restriction. Here, a run with the GibbsCluster excluding indels can cluster only 311 (62%) of the peptides (the remaining peptides are either excluded or placed in the trash cluster) whereas by including indels this number is increased to 467 (94%).

In addition to a more complete picture of the repertoire of naturally presented peptides, the inclusion of ligands of all lengths in the cluster analysis can potentially detect allele-specific preferences in terms of peptide length; such length-profiles are obtained in a fully unsupervised manner and are therefore not affected by potential bias toward certain peptide lengths found in public databases. Beyond the MHC-peptide system, there are several other examples of ligands that bind to their cognate receptor with differentially spaced motifs, as evidenced by the many motifs containing gaps of variable length described in protein motif databases such as ELM (21) and Prosite (22).

The optimal number of clusters in a set of sequences will often depend on the level of resolution desired by the investigator. We have designed GibbsCluster with parameters that allow easy customization of clustering analyses, whether the method should tend to construct small, specialized clusters or larger, more general groups with coarse specificities. A trash cluster can be optionally activated to discard peptides that do not fit into any cluster and in this way detect motifs in noisy sequence data. The sequence motifs identified by the method are displayed in the form of sequence logos and as PSSMs, while alignment and clustering files provide detailed information about the group assignment, scores and alignment core of each peptide. With the GibbsCluster web server we provide a simple and effective tool to analyze peptide datasets and identify multiple receptor-ligand specificities.

## Supplementary Material

Supplementary Data
