# Supplementary material for: GibbsCluster: unsupervised clustering and alignment of peptide sequences
Source: Nucleic Acids Res. 2017 Apr 12;45(Web Server issue):W458–63. doi: 10.1093/nar/gkx248 (PMC5570237; doi:10.1093/nar/gkx248)
Supplement: Supplementary Data [file gkx248_Supp.pdf]

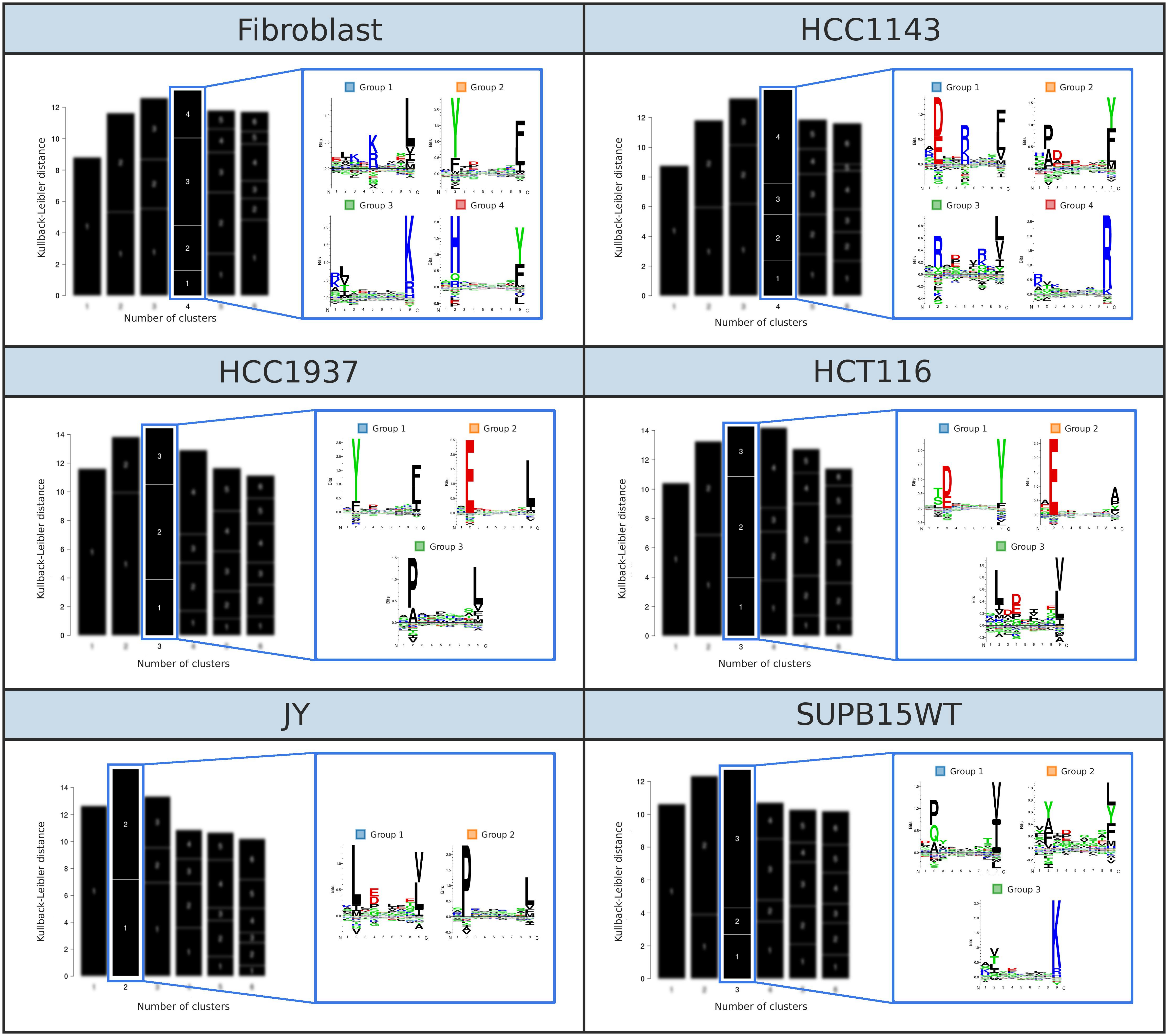

Supplementary Figure 1 *- GibbsCluster output for six different cell lines. Sequence motifs corresponding to the best KLD solutions are shown as sequence logos next to the Kullback-Leibler barplot.*

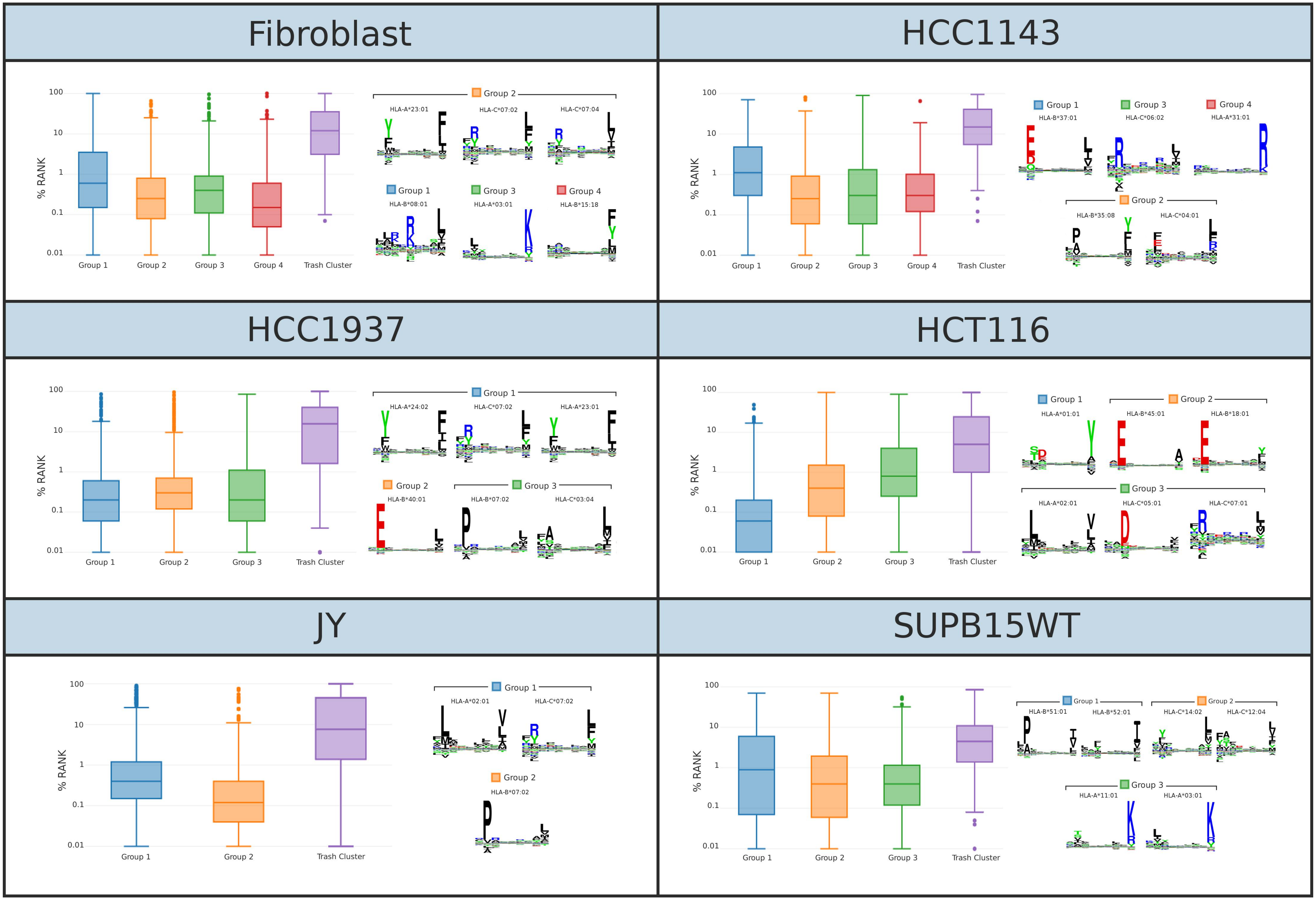

Supplementary Figure 2 *- Solutions of unsupervised clustering compared to HLA restrictions assigned by NetMHCpan for six different cell lines. The left side of each panel shows the distribution of percentile rank scores predicted by NetMHCpan for the allele dominating each cluster; for the trash cluster, the best predicted rank score to any of the six alleles was used. On the right half of each panel are shown the sequence logos from literature of the alleles found in each cluster, generated with MHCcluster (PMID:23775223).*

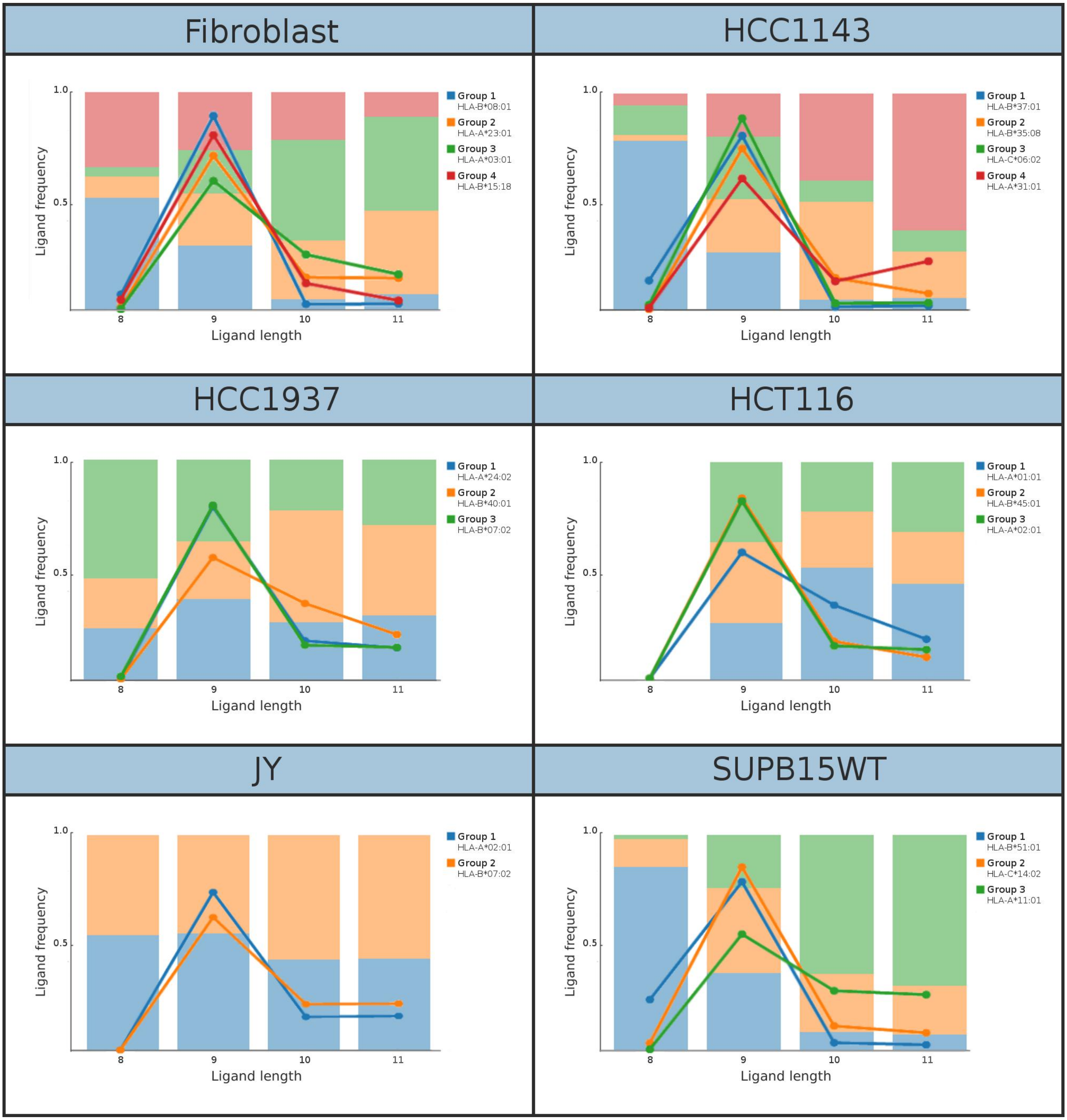

Supplementary Figure 3 *- Binding length profiles of the GibbsCluster solution for six different cell lines. Solid lines depict the percentage of peptides with a given length over the total number of peptides of the group. The stacked barplot in the background represents the length frequency normalized by the height of each column.*
